# Anti-vaccination movements in the world and in Brazil

**DOI:** 10.1590/0037-8682-0592-2021

**Published:** 2022-05-20

**Authors:** Isadora Sousa de Oliveira, Larissa Soares Cardoso, Isabela Gobbo Ferreira, Gabriel Melo Alexandre-Silva, Beatriz de Cássia da Silva Jacob, Felipe Augusto Cerni, Wuelton Marcelo Monteiro, Umberto Zottich, Manuela Berto Pucca

**Affiliations:** 1 Universidade de São Paulo, Faculdade de Ciências Farmacêuticas de Ribeirão Preto, Departamento de Ciências BioMoleculares, Ribeirão Preto, SP, Brasil.; 2 Universidade Federal de Roraima, Faculdade de Medicina, Boa Vista, RR, Brasil.; 3 Universidade Federal de Roraima, Programa de Pós-Graduação em Ciências da Saúde, Boa Vista, RR, Brasil.; 4 Universidade do Estado do Amazonas, Faculdade de Ciências da Saúde, Departamento de Medicina e Enfermagem, Manaus, AM, Brasil.; 5 Fundação de Medicina Tropical Doutor Heitor Vieira Dourado, Departamento de Ensino e Pesquisa, Manaus, AM, Brasil.

**Keywords:** Anti-Vaccination Movement, Anti-Vaxxers, Immunization, Infectious Diseases, Vaccine-Hesitancy

## Abstract

Over the years, vaccinations have provided significant advances in public health, because they substantially reduce the morbimortality of vaccine-preventable diseases. Nevertheless, many people are still hesitant to be vaccinated. Brazil is a region of many anti-vaccine movements, and several outbreaks of vaccine-preventable diseases, such as yellow fever and measles, have occurred in the country during the last few years. To avoid new outbreaks, immunization coverage must be high; however, this is a great challenge to achieve due to the countless anti-vaccine movements. The World Health Organization has suggested new actions for the next decade via the Immunization Agenda 2030 to control, reduce, or eradicate vaccine-preventable diseases. Nonetheless, the vaccination coverage has decreased recently. To resolve the anti-vaccine issue, it is necessary to propose a long-term approach that involves innovative education programs on immunization and critical thinking, using different communication channels, including social media. Cooperation among biology and health scientists, ethicists, human scientists, policymakers, journalists, and civil society is essential for an in-depth understanding of the social action of vaccine refusal and planning effective education measures to increase the vaccine coverage.

## INTRODUCTION

One of the most outstanding achievements in the history of public health is the invention of vaccines, which has contributed to the reduction of the prevalence of many vaccine-preventable diseases (VPDs)^1^. However, since its discovery, vaccination has been a controversial subject. For laypeople, understanding the vaccine mechanism can be complicated. Their concerns include, “*How can a foreign body, created in the laboratory from a pathogen, protect me? What changes occur in my body when the vaccine is given? Would these changes harm other body functions? How can something protect me if it can lead to adverse reactions? How can I be sure that this foreign substance introduced into my body does not carry 'toxins'?*” These and other questions may not have obvious rational answers for those who do not understand the fundamentals of microbiology and immunology.

The rate of childhood vaccination is high in most developed countries, indicating that vaccines remain a widely accepted public health measure. However, the vaccine rates obtained may hide clusters of unvaccinated individuals, and the resurgence of some VPDs has been mainly linked to these under-vaccinated communities^2^ ^,^ ^3^. Experts consider vaccination programs to be threatened by the growing concerns among people. Approximately 5-10% of individuals worldwide have strong anti-vaccination convictions, and a significant proportion is hesitant about vaccination^4^ ^,^ ^5^.

## HISTORY OF ANTI-VACCINATION MOVEMENTS OUTSIDE BRAZIL

Anti-vaccination movements started during the 19^th^ century in England after Edward Jenner introduced vaccination by demonstrating that the cowpox could protect against smallpox^6^ ^,^ ^7^. Furthermore, the Vaccination Act of 1840 in the UK provided free vaccinations to all, then termed as “variolation” (inoculation of smallpox antigens). This act made vaccination compulsory for all children under 14 years and made defaulting parents liable to a fine^8^.

In 1853, vaccination movements boomed after the establishment of an anti*-*vaccination league in London (Table 1)^9^. Subsequently, during the 1870s and 1880s, several anti-vaccination movements started in England, and similar movements flourished all over Europe^9^. The city of Leicester was a particular hotbed of anti-vaccine activities and related demonstrations. Notably, a local newspaper documented, “*An escort was formed, preceded by a banner, to escort a young mother and two men, all of whom had resolved to give themselves up to the police and undergo imprisonment in preference to having their children vaccinated…The three were attended by a numerous crowd…three hearty cheers were given for them, which were renewed with increased vigor as they entered the doors of the police cells.*” The Leicester demonstration occurred in March 1885 and was one of the most notorious anti-vaccine demonstrations in Europe^10^ ^,^ ^11^.

**TABLE 1:** Main anti-vaccination movements outside Brazil.

Year	Local	Event
1853	England	First act against vaccines
		The first anti-vaccination league was created
1879	USA	Anti-vaccination groups were formed
1885	England	Leicester demonstration
1898	England	Movements against the Vaccination Act
1974	England	People hesitance about DTP vaccine
		Reduced vaccination coverage
		Pertussis epidemics
1979-1996	Sweden	Whooping cough vaccination was suspended
1998	England	Movements against MMR vaccine
	USA	Campaigns were made to remove thimerosal and toxins from vaccines
2007	USA	Celebrities supporting anti-vaccine movements

**DTP:** Diphtheria-Tetanus-Pertussis; **MMR:** Measles-Mumps-Rubella.

The growth of anti-vaccine demonstrations led to the development of a commission to study the immunization process. In 1896, the commission proved that vaccination protected against smallpox but suggested the removal of fines for people who refused it. Thus, the Vaccination Act of 1898 removed the fines and included a clause in the law that allowed parents who did not believe in vaccination the right to obtain an exemption certificate^12^.

In the US, smallpox outbreaks led to vaccine campaigns and new anti-vaccine protests consequently. Therefore, the Anti-Vaccination Society of America was founded (1879) following the visit of the British anti-vaccinationist William Tebb. The New England Anti-Compulsory Vaccination League (1882) and Anti-Vaccination League of New York City (1885) were also created, and American anti-vaccinationists waged court battles to repeal vaccination laws in several states, including California, Illinois, and Wisconsin^12^.

In 1902, following a smallpox outbreak, the Board of Health of the city of Cambridge, Massachusetts, made the vaccine against smallpox mandatory for all residents. Henning Jacob refused to receive it, claiming that the law violated his right to make decisions regarding his own body. In response, the city charged him, and after he lost the local court battle, he appealed to the US Supreme Court. In 1905, the court ruled that the state could create compulsory laws to protect the public from infectious diseases. This was the first US Supreme Court case involving a public health law^13^ ^,^ ^14^. 

During the 1970s, international controversy over the safety of the diphtheria-tetanus-pertussis (DTP) vaccine increased in Europe, Asia, Australia, and North America. In the UK, vaccine opposition was the result of a report from the Great Ormond Street Hospital for Sick Children in London, which claimed that 36 children presented neurological problems following DTP immunization^15^. Therefore, vaccination rates decreased even after confirmation of vaccine safety by the Joint Commission on Vaccination and Immunization. Additionally, Gordon Stewart, a physician and vaccine opponent, published many case reports linking neurological disorders to the DTP vaccine, generating additional debates^16^. 

In Sweden, the pertussis vaccine was introduced in the 1950s and withdrawn in 1979 because of concerns about safety and efficacy. Subsequently, no vaccination against pertussis was conducted in Sweden until 1996, leading to approximately 60% children developing the disease before 10 years of age^17^.

In 1998, England was again the center of anti-vaccination activities, presenting movements against the measles, mumps, and rubella (MMR) vaccine. The movement began with Andrew Wakefield, a British physician, who published a study linking the MMR vaccine to autism^18^; however, no other studies have proven this association^19^.

In 1998, the *Green Our Vaccines* campaign started a movement to remove toxins from vaccines, attesting that they lead to autism. One of these toxins is thimerosal, which contains mercury to preserve the vaccine. Although there is no scientific evidence that small amounts of thimerosal in vaccines could be dangerous, it was removed as a precautionary measure^20^. In 2001, the Institute of Medicine’s Immunization Safety Review Committee concluded that there was not enough evidence to prove that thimerosal in childhood vaccines causes autism, attention deficit syndrome, or speech problems^21^. 

Until recently, measles vaccination had largely controlled the outbreaks in the US. In 2013, large measles outbreaks occurred in communities where parents had not vaccinated their children because of philosophical or religious beliefs^22^. The Global Vaccine Action Plan of the World Health Organization (WHO) aims to eliminate measles worldwide; nonetheless, vaccine refusal movements and anti-vaccine programs have interfered with the control of the virus globally. Thus, the measles cases have recently increased to over 700 since January 2019^23^.

Given that the global population has limited scientific knowledge, anti-vaccine movements continue till date. Moreover, movements and demonstrations against vaccines are growing and being supported by many celebrities on social media (celebrity anti-vaxxers), who strongly support Wakefield’s anti-vaccine theory^24^. Thus, it seems that the anti-vaccine movements will not stop. Nevertheless, if the percentage of the vaccinated population continues to decrease, the immunity or resistance to the spread of a disease will fail.

Currently, vaccine hesitancy is considered a movement ranging from refusal of vaccine administration to its delay. Since vaccination acts as a barrier to prevent the transmission of highly contagious diseases, it is crucial to maintain a high rate of population immunization (approximately 95%) to prevent outbreaks^25^. Unfortunately, vaccine hesitance may continue to prevent the world from achieving the desired immunization rate.

## LONG-TERM PROBLEMS OF THE ANTI-VACCINATION MOVEMENTS

Despite a decline in the VPDs, their outbreaks still concern health authorities, and they are important causes of morbimortality associated with reduced vaccination^26^ ^,^ ^27^. For instance, measles has affected European countries and the US during the last 5 years, which may be due to a decrease in the immunization rates due to anti-vaccination movements^28^ ^,^ ^29^. Italy reported over 4,000 measles cases from January to August 2017, and Romania registered approximately 10,000 cases from 2016 to 2017. At the same time, Minnesota experienced a small-scale measles outbreak of 79 cases, which was the largest measles outbreak in the US in the past 30 years^28^ ^-^ ^30^. Measles, a febrile disease with high infectious potential by the respiratory route, typically begins with coryza, conjunctivitis, cough, rash, and fever, and it could evolve into fatal pneumonia and encephalitis^31^. The measles vaccine (MCV) is very effective and frequently administered in two doses, one at 12 months of age (MCV1) and the other at 15-18 months of age (MCV2)^32^, although only one dose is necessary to prevent infection in 99% people^25^.

Vaccination against measles in the US has reduced the number of cases. However, the number of reported measles cases was higher in 2019 than in the previous 25 years^33^ ^,^ ^34^. California also experienced a measles outbreak in 2014 that spread to seven other states, as well as to Canada and Mexico, where the disease affected mostly unvaccinated children, indicating poor vaccination adherence^35^. 

In the last few decades, a decrease in the adherence to immunization programs has been reported worldwide. For example, mumps outbreaks in the Balkans were reported in 2011 due to an interruption in vaccination programs in the 1990s^26^. Over a decade later, mumps-related cases mostly involved non-immunized children, who experienced failures in vaccine coverage from 1992 to 1998 during the Federation of Bosnia and Herzegovina war and post-war period^26^ ^,^ ^36^. In Italy, approximately 90% of emergent measles cases were related to non-immunized children. The age of those affected confirmed poor adherence to vaccination in 1976, when the measles vaccination first started in Italy^28^ ^,^ ^29^. Venezuela is another country that did not have a measles outbreak for many years, though since 2017, it has been suffering from a large-scale epidemic^37^.

Rubella, a highly contagious VPD caused by *Rubivirus*, causes mild symptoms such as fever, adenopathy, and maculopapular rash in children. Additionally, it is responsible for congenital rubella syndrome (CRS), a severe condition associated with deafness, cataract, and cardiac defects in newborns. The occurrence of rubella outbreaks is related to low rubella vaccine coverage, as exemplified by the scenario in Poland. Rubella vaccination started in 1989 in Poland and was restricted to women, resulting in over 21,000 cases in 15-29-year-old men in 2013^26^. Notably, vaccine coverage failures of religious groups lead to the risk of outbreaks, even in countries with high immunization rates, such as polio outbreaks registered in religious clusters in the Netherlands^38^. 

Although the incidence of pertussis reduced after the vaccine’s introduction in the 1940s, the cases have been increasing since 1976^35^. Pertussis or whooping cough is caused by *Bordetella pertussis*, which targets the respiratory system and produces an inflammatory response that leads to paroxysmal cough and cyanosis, mainly in non-immunized patients^39^. Immunized individuals usually develop a mild or asymptomatic version of the disease, but they can still transmit it to non-vaccinated individuals. In the US, there was a cyclical occurrence of the disease, which increased substantially in 2010^40^. This resurgence could be due to multiple reasons, such as greater diagnostic efficacy or replacement of whole-cell vaccines with acellular vaccines^35^. In 2012, approximately 50,000 cases of pertussis occurred in the US in children aged <3 months, who had not yet been immunized^26^. Moreover, countries such as Sweden, Japan, Russia, Ireland, and Italy witnessed a 10-100-fold increase in the incidence of pertussis compared to nations with no reduction in vaccination rates^26^.

Currently, areas of conflict and political unrest are under great threat of outbreaks along with imported cases of VPDs^41^. For instance, a potential outbreak of a wild poliovirus could seriously affect polio-free countries with reduced immunization rates. Hence, the WHO created a plan for the appropriate laboratory containment of possible infectious materials^41^ ^,^ ^42^. Regarding humanitarian emergencies, conflicts in Syria exemplify how political unrest may influence the reemergence of VPDs. Unsanitary conditions and lack of healthcare due to the humanitarian crisis have contributed to rising measles and polio cases, as well as other infections such as leishmaniasis and tuberculosis^43^. Furthermore, the burden of infections may disestablish healthcare and economies worldwide, highlighting the importance of controlling infectious diseases^44^. 

## WHY DO PEOPLE BELIEVE IN THE ANTI-VACCINATION MOVEMENTS?

The increasing number of people who avoid vaccination raises questions about the reasons for this behavior that increases the risk of VPDs^45^. Parents who vaccinate their children are concerned or have doubts regarding vaccine efficacy and risks. Generally, parental decisions regarding vaccination programs are multifactorial and can be divided into individual, group, and contextual categories. The last one can be subdivided into historical, sociocultural, environmental, temporal, institutional, political, and economic reasons, such as lack of health insurance^26^ ^,^ ^46^.

**FIGURE 1:**
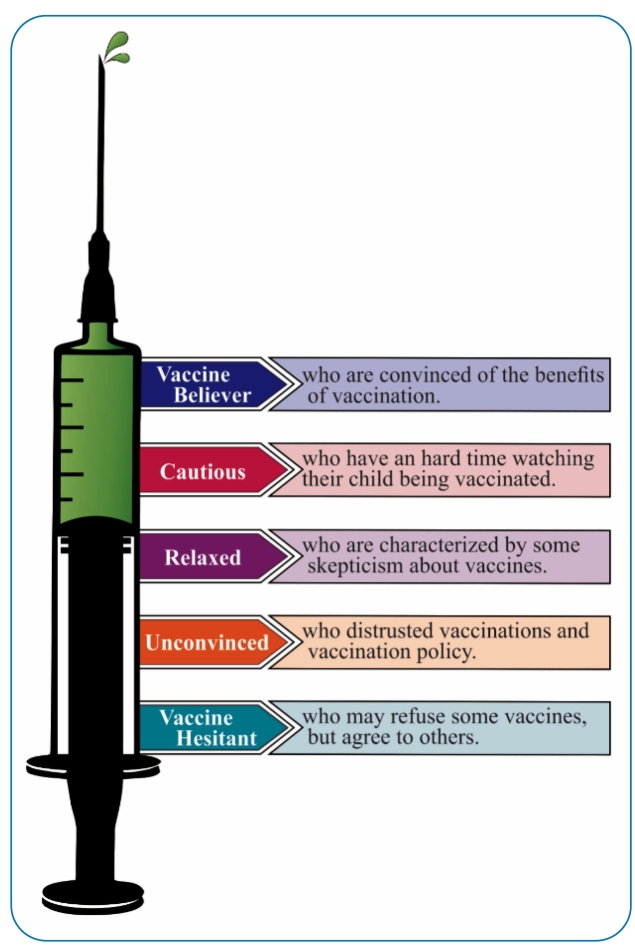
Model of different parent profiles on vaccine acceptance or hesitance.

Vaccination hesitancy has been explored by different models covering acceptance and resistance, most of which focus on parental decision-making. One study identified the following parent profiles: (1) *vaccine believer type*, who are convinced of vaccination benefits; (2) *cautious type*, who are emotionally involved with their child and have a hard time watching them being vaccinated; (3) *relaxed type*, who are skeptical about vaccines; (4) *unconvinced type*, who distrust vaccinations and vaccination policies^47^; and (5) *vaccine-hesitant type,* who are a heterogeneous group that may refuse some vaccines but agree to others (Figure 1). The last group may delay vaccines or accept vaccines according to recommended schedules but may be unsure of their decision^48^.

A WHO study in 2013 regarding vaccination hesitancy demonstrated variability among the 13 studied nations from six WHO regions (Africa, Americas, South East Asia, Europe, Eastern Mediterranean, and Western Pacific)^49^ ^,^ ^50^. The predominant reasons for vaccine hesitancy may vary depending on the socioeconomic status. Emerging/underdeveloped countries lack educational awareness. In developed countries, fear of adverse effects may be greater than beliefs regarding potential benefits^46^. For example, high-income countries no longer have certain infections because of their successful vaccine programs. This “relative” absence of the disease may lead parents to believe in the elimination of infections^26^.

Vaccine safety seems to be an important concern among parents due to studies correlating vaccination to autism and certain potentially dangerous vaccine compounds such as mercury^37^ ^,^ ^49^. Some believe that pharmaceutical companies possess only an economic interest in their products and do not care about risks and adverse effects^49^. Others reported that health professionals do not always explain the potential risks and benefits of the vaccine. However, pediatricians remain the most consulted sources for parents regarding vaccination of their children^26^ ^,^ ^49^.

Psychological studies have shown that those against immunization may have some biases. One is the coincidence bias, wherein any event occurring after vaccination is attributed to it, even though there is no relation^26^. Another is the omission bias, wherein parents prefer the occurrence of a VPD to vaccine-related adverse reactions^26^. Some even justify their negligence saying that the body builds its own immunity^51^. However, adverse reactions are a major concern; parents usually observe fever or soreness after vaccine administration in their children, with rare incidences of serious complications^35^.

Religious reasons have been implicated in vaccine refusal^35^. Some orthodox Protestants believe that the adverse effects experienced by their children after vaccination are a divine punishment. Many religious sects also believe that vaccination interferes with the destiny of humans^52^. Moreover, anti-vaccination and religious movements can be sources of several erroneous concepts. For example, in 2003 in Northern Nigeria, religious leaders and politicians considered vaccination as a tool to induce infertility in Muslims or infect the population with human immunodeficiency virus. This led to a polio resurgence in Nigeria in 2006, resulting in outbreaks in 15 other countries^26^.

Another factor that supports anti-vaccine movements is negative coverage by the media, mainly in Europe and the US^53^. Currently, the link between the MMR vaccine and autism remains a major concern that tends to spread in the media, especially on the Internet, strengthening misinformation^26^. Despite extensive medical literature proving that this association does not exist, parents continue to harbor concerns. There is also concern regarding influenza vaccination and recalcitrant ascending paralysis, known as Guillain-Barré syndrome, although the current vaccine formulation has never been associated with it^54^.

**FIGURE 2:**
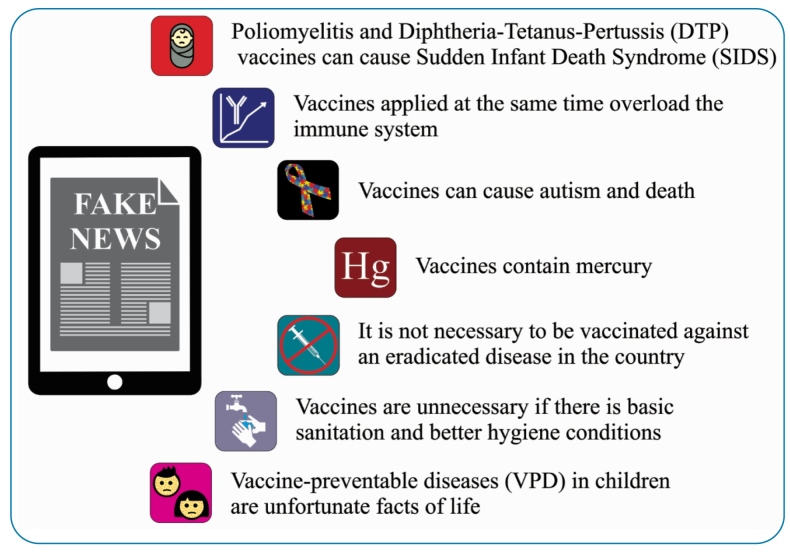
Fake news that may affect acceptance of vaccines.

Social media often spreads many misconceptions about vaccines, known as fake news, usually similar to those already claimed by parents and anti-vaxxers (Figure 2)^55^. Furthermore, websites may appear to be pro-vaccines or use neutral names as a marketing tool for those who seek information, but instead present opinions opposing vaccination to influence parental decisions^26^. Notably, some websites use this strategy along with anti-vaccine quotes of celebrities and politicians, including Jim Carrey, Robert De Niro, Donald Trump, Chuck Norris, Luc Montagnier, and Robert Francis Kennedy Jr.^56^.

Non-immunized adults may contribute to increased occurrence of outbreaks among healthcare workers as well as high-risk groups, such as pregnant women and elderly. The factors that influence hesitancy are variable, including socioeconomic status, religion, media, culture, politics, reliability, and belief in medicine^57^. Some studies on vaccine refusal usually focus on specific vaccines, such as those for influenza or coronavirus disease (COVID-19). Although evidence demonstrates vaccine safety, reduced hospitalizations, and reduced complications, priority groups continue to have low vaccination coverage^58^. Among pregnant women, concerns about the newborn’s safety, autism development in children, and lower educational level are implicated in their decision to not vaccinate^59^ ^,^ ^60^. 

## ANTI-VACCINATION MOVEMENTS IN BRAZIL

Vaccine hesitancy is present worldwide^54^, including Brazil. Over the years, vaccine public programs have been implemented, reformulated, or even discarded against several infectious diseases (*e.g.*, smallpox) in Brazil. However, most vaccines are incapable of overcoming the challenges caused by the physical size of Brazil^61^. One of the most classic reports of vaccine hesitancy in Brazil, which occurred in November 1904, is known as *The Vaccine Revolt*. In that incident, part of the society engaged in physical combat against government agents trying to enforce public health laws and programs^61^ ^,^ ^62^. Although vaccination was mandatory in 1837 for children and in 1846 for adults, the vaccination achieved a significant effect in the country only in 1884^26^ ^,^ ^61^. 

To understand the revolt, we must understand the underlying events that made it possible. Social dissatisfaction is a consequence of a series of events that spread anger and anxiety in the Brazilian population, of which the sanitary reform forcefully implemented in Rio de Janeiro (RJ), misinformation promoted by the media, and physicians who incorrectly performed immunization procedures can be considered the main triggers^12^ ^,^ ^61^ ^,^ ^63^. Thus, the infrastructural remodeling of the cities was motivated mainly by economic and health motives, as tourism was severely affected by diseases. In 1902, agents known as “*Mata-Mosquitos*” (“mosquito-killers”) worked as enforcers to control the *Aedes aegypti* population. In 1902, Oswaldo Cruz, the director of Public Health, granted *Mata-Mosquitos* the authority to invade households to implement control measures. Simultaneously, squads were dedicated to hunting and killing rats to control the bubonic plague, a zoonotic bacterial infection caused by *Yersinia pestis* capable of causing sepsis. These squads also had the authority to invade properties, declare a place unhealthy or inhabitable, and condemn it for demolition, which obviously displeased the occupants^64^ ^-^ ^66^. Before this sanitary reform, RJ was actively avoided by cargo and tourist ships on an international scale due to unhealthy conditions and high incidence of preventable diseases^67^ ^,^ ^68^.

Misinformation and furor reached all layers of society as the media promoted sensationalist and false information to boost their sales. Many physicians participated in this affair by acting outside the field of science or wrongly executing medical procedures and harming patients by trying non-recommended vaccination methods^26^ ^,^ ^61^ ^,^ ^69^. 

During this period, the Society against Mandatory Vaccine, an organized group against vaccines, was created in which people from any social class congregated for a common cause. It was influenced by similar international organizations, such as the Universal Anti-Vaccine League (1885), British National Anti-Vaccination League, and American National Anti-Vaccination League^12^ ^,^ ^63^ ^,^ ^70^.

The previously mentioned laws and government imposition were only a prelude to the National Immunization Plan^71^ devised in 1973 after the sequential success of the Smallpox Eradication Campaign under Oswaldo Cruz and the National Control Plan for Poliomyelitis (1971-1973).

With the recent emergence of the COVID-19 pandemic, health authorities have recommended aggressive implementation of suppression strategies, such as case identification, quarantine and isolation, contact tracing, and social distancing. Mathematical models have demonstrated that the COVID-19 spread can rebound quickly if these interventions are relaxed^72^. Therefore, high vaccination coverage with safe and effective vaccines globally is a powerful public health measure^73^. Although scientists, politicians, and leaders from different countries have fought a real race for the “most expected vaccine in history,”^74^ a misinformation bubble has threatened the vaccine campaigns. Currently, Brazilian scientific and civil groups are acting against delayed vaccination to mitigate the pandemic effects.

## OUTBREAKS IN BRAZIL

With the vaccine introduction in Brazil, reduction or elimination of pertussis, diphtheria, polio, tuberculosis, yellow fever (YF), and smallpox has become possible^75^. To control these VPDs, a high rate of vaccination coverage is imperative, especially considering the global travel to emerging countries. However, with the decrease in vaccinations and increase in immigrants, an environment conducive for outbreaks has been created in Brazil^76^.

### Measles

Although the measles vaccination was implemented in Brazil in 1960, its recurrence was prevented only in 2000, with over 95% of the population immunized^77^. 

In Brazil, between 2000 and 2017, MCV prevented 21.1 million deaths. Even with high adherence rates to the vaccine, 3-7% of the population remains susceptible to the disease, and outbreaks still occur^78^. An outbreak was reported in 1997, and most of the affected individuals were adults. During 2001-2013, the majority of measles cases in the country were imported, *i.e.*, individuals contracted measles outside Brazil, unlike the outbreak that occurred in Ceará state during 2013-2014, wherein transmission occurred locally^79^ ^,^ ^80^.

**FIGURE 3:**
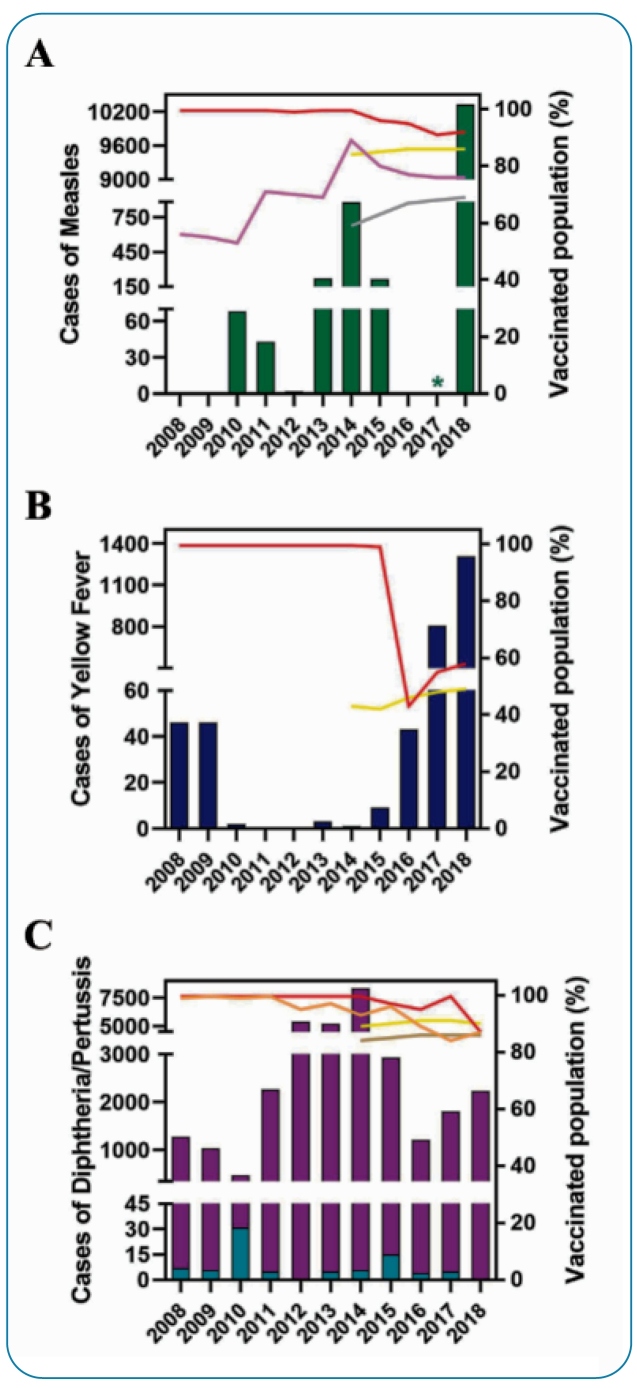
Reported cases of outbreaks and their vaccination coverage in Brazil (2008-2018)**. (A)** Cases of measles: Green bars indicate measles cases. **(B)** Cases of yellow fever: Dark blue bars indicate yellow fever cases. **(C)** Cases of diphtheria and pertussis: Light blue bars indicate diphtheria cases, and purple bars indicate pertussis cases. Red and yellow lines indicate percentage of the Brazilian and global population, respectively, vaccinated with the first/single dose of the corresponding vaccine. Pink line indicates percentage of the Brazilian population vaccinated with the second dose of measles vaccine (MCV2). Orange line indicates percentage of the Brazilian population vaccinated with the third dose of DTP vaccine (DTP3). Gray and brown lines indicate percentage of global population vaccinated with MCV2 and DTP3, respectively. Data were obtained from *Ministério da Saúde do Brasil* ^88^ and WHO^34^ (*Not reported).

In 2016, the country received a WHO certificate recognizing the end of the circulation of measles virus^77^. Unfortunately, another outbreak occurred in Brazil in 2018, in which over 10,000 cases were identified, most of which were in the states of Amazonas and Roraima, as well as in São Paulo, Rio Grande do Sul, Pernambuco, RJ, Sergipe, and Pará. One contributing factor was the importation of the measles D8 genotype that arrived via Venezuelan immigrants. Moreover, the population is less adherent to MCV1 and even lesser to MCV2; thus, most of the population is not vaccinated. However, the percentage of the Brazilian vaccinated population is higher than that of the globally vaccinated population against measles (Figure 3A)^34^ ^,^ ^76^ ^,^ ^79^.

## YELLOW FEVER

YF is caused by a virus of *Flavivirus* genus. Its transmission cycles include transmission by the main vector, *A. aegypti* (urban YF), and transmission by mosquitoes of the *Haemagogus* genus (wild YF), which is mostly related to occurrence in primates. YF is a febrile disease characterized by acute infection of short duration. It can evolve into severe forms, causing liver and kidney failure, which often lead to death^81^. 

The largest urban YF epidemic occurred in Brazil during 1928-1929; no additional cases were recorded after 1942. The main measures taken to control YF were mass vaccination and vector control, which guaranteed interruption of the disease transmission cycle for many years. However, in 2002, there was an outbreak in Minas Gerais state that affected two or three municipalities. During 2008-2009, an increasing number of cases was observed, spreading the virus throughout South, Southeast, and Midwest Brazil. In 2017, another outbreak occurred in Minas Gerais, affecting 90 cities, and in other Brazilian states, such as Distrito Federal, Espírito Santo, Goiás, Mato Grosso, Pará, RJ, São Paulo, and Tocantins^82^ ^-^ ^85^. Since only 58% of the Brazilian population was immunized in 2018, reduced vaccination coverage was considered the main cause of these outbreaks (Figure 3B)^34^.

### Diphtheria and pertussis

Diphtheria is caused by *Corynebacterium diphtheriae* toxins, which cause inflammation of nasopharyngeal membranes, fever, and cough. It is highly contagious and has a high mortality rate in children. The DTP vaccine was first introduced in Brazil in 1973. In 2010, a diphtheria outbreak occurred in Maranhão state. As there are regions in the world where the disease remains endemic with frequent outbreaks, such as Venezuela, the arrival of immigrants can lead to disease recurrence in specific areas. However, in 2016, coverage of the first dose of DTP vaccine was 95%, and that of the third dose was 89%, leading to a significant decrease in incidence. Between 2013 and 2017, only 36 cases were registered in Brazil^34^ ^,^ ^86^ ^-^ ^88^.

The incidence of pertussis has decreased since the beginning of DTP vaccination, especially in 1980. However, outbreaks occurred in the Midwest region between 2012 and 2014, with over 4,000 cases/year^34^ ^,^ ^89^. Pertussis outbreaks are related to phenotypic changes in bacterial strains, reduced adherence to vaccination, and reduced potential to induce immunity^89^ (Figure 3C).

### Others

Polio majorly affects children aged <5 years; approximately 20 million people worldwide live with the consequences of polio, i.e., paralysis^90^. In the 1980s, there were approximately 3,000 polio cases in Brazil, and an extensive vaccination campaign against polio resulted in its eradication in the 1990s^34^ ^,^ ^91^.

In Brazil, there have been several rubella outbreaks between 1991 and 2001, there was a 95% decrease in cases in 2002 from that in 1997. New outbreaks were registered between 2005 and 2007, leading to a vaccination campaign in 2008 that reached 96.7% of the target population. Since then, cases decreased until 2015, when the elimination of rubella was reported, and Brazil received the rubella and CRS elimination certificate in 2015^92^ ^,^ ^93^.

## NEW ERA OF ANTI-VACCINATION MOVEMENTS DURING COVID-19 PANDEMIC

COVID-19 has already affected millions of people and led to the mortality of thousands worldwide^94^. However, there is no effective treatment established yet for COVID-19. Hence, its prevention through highly effective and widely distributed vaccines is the most promising approach^95^.

Many vaccines against COVID-19 have been approved and used till date^96^. This biggest and most ambitious vaccination campaigns in history led to a decrease in COVID-19 cases and hospitalizations^97^. Unfortunately, the administration of vaccines is suboptimal; the ongoing VPD resurgence led the WHO to consider vaccine hesitancy in the top 10 threats to global health^98^. 

Vaccine hesitancy may be fueled by various opinions regarding vaccination, from cautious users to outright deniers^99^. Among the barriers to universal vaccination, misinformation regarding its benefits, medicinal composition, and adverse effects limit patient understanding and overall acceptance^100^. 

The COVID-19 vaccines developed are currently being used worldwide; despite no long-term studies, they are considered safe and the best alternative to break the viral transmission^101^. Notably, most of them have only caused mild adverse reactions (redness, swelling, muscle pain, and fever), and their efficacy has been proven in clinical trials^102^ ^,^ ^103^. To date, over 50% of the global population (4.07 billion) has been fully vaccinated^104^, resulting in a significant decrease in transmission and mortality.

In conclusion, although vaccine coverage has eradicated or controlled many infectious diseases worldwide, the coverage proportion has reduced over the last few years, suggesting that anti-vaccine movements affect coverage rates. For most individuals, vaccine hesitancy is frequently motivated by influential misperceptions of the vaccine risks. Hence, we urgently need a long-term approach to overcome vaccine hesitancy that involves educating people regarding immunization and critical thinking, using different communication channels including social media. 

## References

[1] Fine P, Eames K, Heymann DL (2011). “Herd Immunity”: A Rough Guide. Clin Infect Dis.

[2] Falagas ME, Zarkadoulia E (2008). Factors Associated with Suboptimal Compliance to Vaccinations in Children in Developed Countries: A Systematic Review. Curr Med Res Opin.

[3] Omer SB, Salmon DA, Orenstein WA, deHart MP, Halsey N (2009). Vaccine Refusal, Mandatory Immunization, and the Risks of Vaccine-Preventable Diseases. N Engl J Med.

[4] Black S, Rappuoli R (2010). A Crisis of Public Confidence in Vaccines. Sci Transl Med.

[5] Leask J, Braunack-Mayer A, Kerridge I (2011). Consent and Public Engagement in an Era of Expanded Childhood Immunisation. J Paediatr Child Health.

[6] Grignolio A (2018). A Brief History of Anti-Vaccination Movements. In Grignolio A, editor. Vaccines: Are they Worth a Shot?.

[7] Barquet N, Domingo P (1997). Smallpox: The Triumph over the Most Terrible of the Ministers of Death. Ann Intern Med.

[8] Mariner WK, Annas GJ, Glantz LH (2005). Jacobson v Massachusetts: It’s Not Your Great-Great-Grandfather’s Public Health Law. Am J Public Health.

[9] Porter D, Porter R (1988). The Politics of Prevention: Anti-Vaccinationism and Public Health in Nineteenth-Century England. Med Hist.

[10] Hussain A, Ali S, Ahmed M, Hussain S (2018). The Anti-Vaccination Movement: A Regression in Modern Medicine. Cureus.

[11] Durbach N (2000). “They Might as Well Brand Us”: Working-Class Resistance to Compulsory Vaccination in Victorian England. Soc Hist Med.

[12] Wolfe RM, Sharp LK (2002). Anti-Vaccinationists Past and Present. BMJ.

[13] Gostin LO (2005). Jacobson v Massachusetts at 100 Years: Police Power and Civil Liberties in Tension. Am J Public Health.

[14] Albert MR, Ostheimer KG, Breman JG (2001). The Last Smallpox Epidemic in Boston and the Vaccination Controversy, 1901-1903. N Engl J Med.

[15] Kulenkampff M, Schwartzman JS, Wilson J (1974). Neurological Complications of Pertussis Inoculation. Arch Dis Child.

[16] Baker JP (2003). The Pertussis Vaccine Controversy in Great Britain, 1974-1986. Vaccine.

[17] Hallander HO, Advani A, Donnelly D, Gustafsson L, Carlsson RM (2005). Shifts of Bordetella Pertussis Variants in Sweden from 1970 to 2003, during Three Periods Marked by Different Vaccination Programs. J Clin Microbiol.

[18] Wakefield AJ, Murch SH, Anthony A, Linnell J, Casson DM, Malik M (2010). Retraction--Ileal-Lymphoid-Nodular Hyperplasia, Non-Specific Colitis, and Pervasive Developmental Disorder in Children. Lancet.

[19] Stratton K, Gable A, Shetty P, McCormick M, Institute of Medicine (US) Immunization Safety Review Committee (2001). Immunization Safety Review: Measles-Mumps-Rubella Vaccine and Autism.

[20] (2013). Understanding Thimerosal, Mercury, and Vaccine Safety.

[21] Stratton K, Gable A, McCormick MC, Institute of Medicine (US) Immunization Safety Review Committee (2001). Immunization Safety Review: Thimerosal-Containing Vaccines and Neurodevelopmental Disorders.

[22] Bester JC (2016). Measles and Measles Vaccination: A Review. JAMA Pediatr.

[2] Porter A, Goldfarb J (2019). Measles: A Dangerous Vaccine-Preventable Disease Returns. CCJM.

[24] Gross L (2009). A Broken Trust: Lessons from the Vaccine--Autism Wars. PLoS Biol.

[25] Fine PE (1993). Herd Immunity: History, Theory, Practice. Epidemiol Rev.

[26] Dubé E, Vivion M, MacDonald NE (2015). Vaccine Hesitancy, Vaccine Refusal and the Anti-Vaccine Movement: Influence, Impact and Implications. Expert Rev Vaccines.

[27] Briand SC (2016). Into the Future: Are We Ready to Face Modern Outbreaks?. Wkly Epidemiol Rec.

[28] Orosz L, Gáspár G, Rózsa Á, Rákos N, Sziveri S, Bosnyákovits T (2018). Epidemiological Situation of Measles in Romania, Italy, and Hungary: On What Threats Should We Focus Nowadays?. Acta microbiol immunol Hung.

[29] Filia A, Bella A, Del Manso M, Baggieri M, Magurano F, Rota MC (2017). Ongoing Outbreak with Well over 4,000 Measles Cases in Italy from January to End August 2017 - What Is Making Elimination so Difficult?. Euro Surveill.

[30] Leslie TF, Delamater PL, Yang YT (2018). It Could Have Been Much Worse: The Minnesota Measles Outbreak of 2017. Vaccine.

[31] Moss WJ (2017). Measles. Lancet.

[32] World Health Organization (WHO) (2019). Table 1: Summary of WHO Position Papers - Recommendations for Routine Immunization.

[33] Davis MM, Shah SK (2019). Outbreaks of Vaccine-Preventable Diseases: Responding to System Failure With National Vaccination Requirements. JAMA.

[34] World Health Organization (WHO) (2020). Immunization Country Profile.

[35] Phadke VK, Bednarczyk RA, Salmon DA, Omer SB (2016). Association Between Vaccine Refusal and Vaccine-Preventable Diseases in the United States. JAMA.

[36] Hukic M, Ravlija J, Ljubovic AD, Moro A, Arapcic S, Muller CP (2011). Ongoing Large Mumps Outbreak in the Federation of Bosnia and Herzegovina, Bosnia and Herzegovina, December 2010 to July 2011. Euro Surveill.

[37] Paules CL, Marston HD, Fauci AS (2019). Measles in 2019 - Going Backward. N Engl J Med.

[38] Oostvogel PM, van Wijngaarden JK, van der Avoort HG, Mulders MN, Conyn-van Spaendonck MA, Rümke HC (1994). Poliomyelitis Outbreak in an Unvaccinated Community in the Netherlands, 1992-93. Lancet.

[39] Nguyen VTN, Simon L (2018). Pertussis: The Whooping Cough. Prim Care.

[40] Winter K, Harriman K, Schechter R, Yamada E, Talarico J, Chavez G (2020). Notes from the Field: Pertussis - California, January-June 2010.

[41] Martin N, Paterson BJ, Durrheim DN (2014). Australia’s Polio Risk. Commun Dis Intell Q Rep.

[42] World Health Organization (WHO) (2003). WHO Global Action Plan for Laboratory Containment of Wild Polioviruses.

[43] Ozaras R, Leblebicioglu H, Sunbul M, Tabak F, Balkan II, Yemisen M (2016). The Syrian Conflict and Infectious Diseases. Expert Rev Anti Infect Ther.

[44] Nii-Trebi NI (2017). Emerging and Neglected Infectious Diseases: Insights, Advances, and Challenges. Biomed Res Int.

[45] Czajka H (2018). Why are preventive vaccinations still required?. Dev Period Med.

[46] Lane S, MacDonald NE, Marti M, Dumolard L (2018). Vaccine Hesitancy around the Globe: Analysis of Three Years of WHO/UNICEF Joint Reporting Form Data-2015-2017. Vaccine.

[47] Keane MT, Walter MV, Patel BI, Moorthy S, Stevens RB, Bradley KM (2005). Confidence in Vaccination: A Parent Model. Vaccine.

[48] Opel DJ, Mangione-Smith R, Taylor JA, Korfiatis C, Wiese C, Catz S (2011). Development of a Survey to Identify Vaccine-Hesitant Parents: The Parent Attitudes about Childhood Vaccines Survey. Hum Vaccin.

[49] Giambi C, Fabiani M, D’Ancona F, Ferrara L, Fiacchini D, Gallo T (2018). Parental Vaccine Hesitancy in Italy - Results from a National Survey. Vaccine.

[50] World Health Organization (WHO) (2020). Definition of regional groupings.

[51] Harmsen IA, Mollema L, Ruiter RAC, Paulussen TGW, de Melker HE, Kok G (2013). Why Parents Refuse Childhood Vaccination: A Qualitative Study Using Online Focus Groups. BMC Public Health.

[52] Ruijs WLM, Hautvast JLA, van Ijzendoorn G, van Ansem WJC, van der Velden K, Hulscher MEJL (2012). How Orthodox Protestant Parents Decide on the Vaccination of Their Children: A Qualitative Study. BMC Public Health.

[53] Larson HJ, Jarrett C, Schulz WS, Chaudhuri M, Zhou Y, Dube E (2015). Measuring Vaccine Hesitancy: The Development of a Survey Tool. Vaccine.

[54] Callender D (2016). Vaccine Hesitancy: More than a Movement. Hum Vaccin Immunother.

[55] Ministério da Saúde do Brasil (2020). Vacinação: quais são as vacinas, para quê servem, por que vacinar, mitos.

[56] Arif N, Al-Jefri M, Bizzi IH, Perano GB, Goldman M, Haq I (2018). Fake News or Weak Science? Visibility and Characterization of Antivaccine Webpages Returned by Google in Different Languages and Countries. Front Immunol.

[57] Larson HJ, Jarrett C, Eckersberger E, Smith DMD, Paterson P (2014). Understanding Vaccine Hesitancy around Vaccines and Vaccination from a Global Perspective: A Systematic Review of Published Literature, 2007-2012. Vaccine.

[58] Pinto CJM, Pereira EHR, Teodoro CM, Becari RA, Assis VG, Ferrari JC (2019). Vaccination against Influenza in Elderly People: Factors Associated with Acceptance and Refusal of the Vaccine. Rev Soc Bras Med Trop.

[59] Descamps A, Launay O, Bonnet C, Blondel B (2019). Seasonal Influenza Vaccine Uptake and Vaccine Refusal among Pregnant Women in France: Results from a National Survey. Hum Vaccin Immunother.

[60] OʼLeary ST, Riley LE, Lindley MC, Allison MA, Albert AP, Fisher A (2019). Obstetrician-Gynecologists’ Strategies to Address Vaccine Refusal Among Pregnant Women. Obstet Gynecol.

[61] Fernandes T (1999). Vacina Antivariólica: Seu Primeiro Século No Brasil (Da Vacina Jenneriana à Animal). História, Ciências, Saúde-Manguinhos.

[62] Pôrto Â, Ponte CF (2003). Vacinas e Campanhas: As Imagens de Uma História a Ser Contada. História, Ciências, Saúde-Manguinhos.

[63] Lopes MB (2001). O Rio Em Movimento: Quadros Médicos e(m) História 1890 - 1920.

[64] OUR MAGAZINE COVER (2002). Oswaldo Cruz. Jornal Brasileiro de Patologia e Medicina Laboratorial.

[65] da Silva Magalhães RC (2016). A Erradicação Do Aedes Aegypti: Febre Amarela, Fred Soper e Saúde Pública Nas Américas (1918-1968).

[66] Perry RD, Fetherston JD (1997). Yersinia Pestis--Etiologic Agent of Plague. Clin Microbiol Ver.

[67] Britto N (1995). Oswaldo Cruz: A Construção de Um Mito Na Ciência Brasileira.

[68] Meade T (1986). “Civilizing Rio de Janeiro”: The Public Health Campaign and the Riot of 1904. J Soc Hist.

[69] Schwartz JL (2012). New Media, Old Messages: Themes in the History of Vaccine Hesitancy and Refusal. Virtual Mentor.

[70] (2022). United Statesian.

[71] Domingues CMAS, Teixeira AMS (2013). Coberturas Vacinais e Doenças Imunopreveníveis No Brasil No Período 1982-2012: Avanços e Desafios Do Programa Nacional de Imunizações. Epidemiol Serv Saúde.

[72] Ferguson N, Laydon D, Nedjati Gilani G, Imai N, Ainslie K, Baguelin M (2020). Report 9: Impact of Non-Pharmaceutical Interventions (NPIs) to Reduce COVID19 Mortality and Healthcare Demand.

[73] Yamey G, Schäferhoff M, Hatchett R, Pate M, Zhao F, McDade KK (2020). Ensuring Global Access to COVID-19 Vaccines. Lancet.

[74] Paula JE, Camilo LP, Siqueira EW (2021). The Race for Vaccination against Covid-19 in Brazil: How Can the Fake News and the Negligence of the Government Influence the Immunization?.

[75] Braz RM, Domingues CMAS, Teixeira AMS, Luna EJA (2016). Classification of Transmission Risk of Vaccine-Preventable Diseases Based on Vaccination Indicators in Brazilian Municipalities. Epidemiol Serv Saúde.

[76] Fujita DM, Salvador FS, Nali LHS, Luna EJA (2018). Decreasing Vaccine Coverage Rates Lead to Increased Vulnerability to the Importation of Vaccine-Preventable Diseases in Brazil. J Travel Med.

[77] Almeida CCC, Carvalho GB, Ferreira JS, Souza LVG, Moura Fé MS, Fontenele APS (2020). Estudo Epidemiológico de Pacientes Infectados Por Sarampo No Brasil. BJHR.

[78] Almeida CMS, Souza LGD, Coelho GN, Almeida KC (2020). Correlação Entre o Aumento Da Incidência de Sarampo e a Diminuição Da Cobertura Vacinal Dos Últimos 10 Anos No Brasil. BJHR.

[79] Goldani LZ (2018). Measles Outbreak in Brazil, 2018. Braz J Infect Dis.

[80] Fonnesbeck CJ, Shea K, Carran S, Cassio de Moraes J, Gregory C, Goodson JL (2018). Measles Outbreak Response Decision-Making under Uncertainty: A Retrospective Analysis. J R Soc Interface.

[81] Cavalcante KRLJ, Tauil PL (2016). Características epidemiológicas da febre amarela no Brasil, 2000-2012. Epidemiol Serv Saúde.

[82] Cavalcante KRLJ, Tauil PL (2017). Risk of Re-Emergence of Urban Yellow Fever in Brazil. Epidemiol Serv Saúde.

[83] Rossetto EV, Angerami RN, Luna EJA (2017). What to Expect from the 2017 Yellow Fever Outbreak in Brazil?. Rev Inst Med Trop Sao Paulo.

[84] Possas C, Lourenço-de-Oliveira R, Tauil PL, Pinheiro FP, Pissinatti A, Cunha RV (2018). Yellow Fever Outbreak in Brazil: The Puzzle of Rapid Viral Spread and Challenges for Immunisation. Memórias do Instituto Oswaldo Cruz.

[85] Pan American Health Organization; World Health Organization (2017). Epidemiological Update: Yellow Fever.

[86] Vieira S, Meira AMM, Carvalho AL, Nepomuceno IA, Diniz LMO, Romanelli RMC (2019). Atualização Em Difteria. Rev Med Minas Gerais.

[87] Santos CA (2019). Cenário Epidemiológico Da Difteria Na Atualidade.

[88] Ministério da Saúde (MS) (2021). DATASUS - Doenças e Agravos de Notificação.

[89] Rocha EL, Leite D, Camargo CH, Martins LM, Silva RSN, Martins VP (2017). The Characterization of Bordetella Pertussis Strains Isolated in the Central-Western Region of Brazil Suggests the Selection of a Specific Genetic Profile during 2012-2014 Outbreaks. Epidemiol Infect.

[90] Jones KM, Balalla S, Theadom A, Jackman G, Feigin VL (2017). A Systematic Review of the Worldwide Prevalence of Survivors of Poliomyelitis Reported in 31 Studies. BMJ Open.

[91] Ministério da Saúde (MS) (2020). Poliomielite: causas, sintomas, diagnóstico e prevenção.

[92] Ministério da Saúde (MS) (2020). Rubéola: quais os sintomas, como é transmitida e como prevenir.

[93] Ludert JE, Pujol FH, Arbiza J (2017). Human Virology in Latin America: From Biology to Control.

[94] World Health Organization (WHO) (2020). WHO COVID-19 Dashboard.

[95] Lu H (2020). Drug Treatment Options for the 2019-New Coronavirus (2019-NCoV). Biosci Trends.

[96] Yadav T, Srivastava N, Mishra G, Dhama K, Kumar S, Puri B (2020). Recombinant Vaccines for COVID-19. Hum Vaccin Immunother.

[97] Chung JY, Thone MN, Kwon YJ (2021). COVID-19 Vaccines: The Status and Perspectives in Delivery Points of View. Adv Drug Deliv Rev.

[98] World Health Organization (WHO) (2022). Ten health issues WHO will tackle this year.

[99] Carrieri V, Madio L, Principe F (2019). Vaccine Hesitancy and (Fake) News: Quasi-Experimental Evidence from Italy. Health Econ.

[100] Tustin JL, Crowcroft NS, Gesink D, Johnson I, Keelan J, Lachapelle B (2018). User-Driven Comments on a Facebook Advertisement Recruiting Canadian Parents in a Study on Immunization: Content Analysis. JMIR Public Health Surveill.

[101] Marian AJ (2021). Current State of Vaccine Development and Targeted Therapies for COVID-19: Impact of Basic Science Discoveries. Cardiovasc Pathol.

[102] Han X, Xu P, Ye Q (2021). Analysis of COVID-19 Vaccines: Types, Thoughts, and Application. J Clin Lab Anal.

[103] Al-Kassmy J, Pedersen J, Kobinger G (2020). Vaccine Candidates against Coronavirus Infections. Where Does COVID-19 Stand?. Viruses.

[104] Ritchie H, Mathieu E, Rodés-Guirao L, Appel C, Giattino C, Ortiz-Ospina E (2022). Coronavirus Pandemic (COVID-19). Our World.

